# Improving the predictive power of xenograft and syngeneic anti-tumour studies using mice humanised for pathways of drug metabolism

**DOI:** 10.12688/f1000research.122987.1

**Published:** 2022-09-21

**Authors:** Colin J. Henderson, Aileen W. McLaren, Yury Kapelyukh, C. Roland Wolf

**Affiliations:** 1 Division of Systems Medicine, School of Medicine, University of Dundee, Ninewells Hospital, Dundee, Tayside, DD1 9SY, UK

**Keywords:** humanised P450 model, transgenic mice, drug metabolism, drug development, xenograft, chemotherapy, combination chemotherapy

## Abstract

Drug development is an expensive and time-consuming process, with only a small fraction of drugs gaining regulatory approval from the often many thousands of candidates identified during target validation. Once a lead compound has been identified and optimised, they are subject to intensive pre-clinical research to determine their pharmacodynamic, pharmacokinetic and toxicological properties, procedures which inevitably involve significant numbers of animals - mainly mice and rats, but also dogs and monkeys in much smaller numbers and for specific types of drug candidates. Many compounds that emerge from this process, having been shown to be safe and efficacious in pre-clinical studies, subsequently fail to replicate this outcome in clinical trials, therefore wasting time, money and, most importantly, animals.

The poor predictive power of animal models in pre-clinical studies is predominantly due to lack of efficacy or safety reasons, which in turn can be attributed mainly to the significant species differences in drug metabolism between humans and animals. To circumvent this, we have developed a complex transgenic mouse model – 8HUM - which faithfully replicates human Phase I drug metabolism (and its regulation), and which will generate more human-relevant data [ *REFINEMENT*] from fewer animals [ *REDUCTION*] in a pre-clinical setting and reduce attrition in the clinic.

One key area for the pre-clinical application of animals in an oncology setting – almost exclusively mice - is their use in anti-tumour studies. We now further demonstrate the utility of the 8HUM mouse using a murine melanoma cell line as a syngeneic tumour and also present an immunodeficient version 8HUM_Rag2 ^-/-^ - for use in xenograft studies. These models will be of significant benefit not only to Pharma for pre-clinical drug development work, but also throughout the drug efficacy, toxicology, pharmacology, and drug metabolism communities, where fewer animals will be needed to generate more human-relevant data.

## Abbreviations

PDX: patient-derived xenograft

p.o.: per os, oral gavage

s.c.: sub-cutaneous

## Introduction

The pre-clinical stage of drug development provides crucial information for the decision process as to whether a drug candidate will proceed to ‘first in human’ and phased clinical trials. ^1^ The failure rate through the pre-clinical stages of drug development can be high and many candidate molecules taken forward to clinical trials subsequently fail to recapitulate the safety profile and efficacy found in animal studies. ^2^^–^^5^ There are many reasons for this, but significant species differences in drug metabolism between animals (rats, mice) and humans – with concomitant changes in pharmacokinetics, metabolite profiles, toxicokinetics and pharmacodynamics – are key components underlying the observed failure rates. ^5^ We have developed a sophisticated transgenic mouse model in which the major human drug metabolising enzymes – and the transcription factors regulating their expression – replace their mouse counterparts. In a previous report on this humanised mouse model ^6^ we show, using model compounds and anti-cancer drugs, that drug metabolism and disposition in the 8HUM mouse more closely reflects that found in humans. Given the growing importance of drug combinations in cancer therapy, ^7^ it is clear that a genetically engineered mouse model such as 8HUM could play a pivotal role in the development of such combinations. ^8^

While some pre-clinical work is carried out *in vitro*, using a variety of cell lines including immortalised human cells, much – and arguably the most important – is carried out in animals, mainly rodents but also dogs, and for certain types of candidate molecules, primates. One such *in vivo* use in the pre-clinical setting is syngeneic or xenograft work, where anti-tumour efficacy of drug candidates is tested alone or in drug combinations. In a syngeneic model, murine cell lines are implanted subcutaneously or orthotopically and tumour response to candidate drugs tested. Whilst such experiments can account for the effect of immune system, the genetic background of the tumour cells - which must match that of the host animal used – can also give rise to disparate results. More recently xenograft models have come to the fore, where immunodeficient mouse lines are able to grow human tumours either from existing immortalised cell lines or via fresh tissue as patient-derived xenografts (PDX). ^9^^,^^10^ Despite lack of a competent immune system and any issues that this may potentially cause in the interpretation of results, the latter are growing in use, a good example being the EurOPDX consortium, who have a database of PDX models to share with the research community. ^11^ Notwithstanding extensive xenograft use in various guises, such models still have issues arising from retention of murine drug metabolism and disposition. ^8^

In this brief report we showcase a modification of the humanised 8HUM model in which we have generated a compromised immune system by deleting the *Rag2* locus. Using murine and human melanoma cell lines in 8HUM_Rag2 ^-/-^ mice, we show tumour growth in a syngeneic and xenograft setting, respectively, and demonstrate *in vivo* sensitivity to dabrafenib and trametinib, drugs currently used in combination as standard of care in the treatment of metastatic melanoma. Together, these models have the potential to significantly reduce the number of animals used in the pre-clinical stages of drug development, while generating more human-relevant data and thus improving the chances of a candidate drug replicating a positive pre-clinical finding in successful clinical trials.

## Methods

### Reagents

Unless specifically stated, reagents used in these studies were purchased from Sigma-Aldrich (Dorset, UK).

### Animals

Transgenic mice – 8HUM - extensively humanized for the major cytochrome P450 enzymes in Phase I drug metabolism, along with the transcription factors regulating their expression, have previously been described ^6^ and were generated in a collaboration between CXR Biosciences and Taconic Biosciences funded through the Scottish Government ITI, with CRW as one of the principal investigators.

Thirty-five murine genes (the Cyp2c (except Cyp2c44), Cyp2d and Cyp3a murine gene clusters and transcription factors Car and Pxr) were replaced by eight human genes (CYP1A1, CYP1A2, CYP2C9, CYP2D6, CYP3A4, CYP3A7, CAR, PXR). Expression of human P450 genes was from the human promotor, except for CYP2C9, which was driven by the albumin promotor, and CYP1A1, CYP1A2, CAR and PXR which were driven off the corresponding murine promoters.

8HUM mice were further genetically altered by deleting *Rag2* using CRISPR/cas9-mediated gene editing in 8HUM zygotes (Taconic Biosciences GmbH, Germany). Breeding of mice from this process was carried out to re-generate 8HUM mice with a homozygous deletion of *Rag2*-8HUM_Rag2 ^-/-^-rendering the line immunodeficient for xenograft studies.

Animals were on a C57BL/NTac background and were bred, and experimental work carried out in the Medical School Resource Unit, University of Dundee. Mice were held at positive pressure in Techniplast Sealsave BlueLine micro-isolator cages, with Eco-Pure chip7D bedding (Datesand Group, UK) and *ad libitum* access to water and food-RM1 for maintenance, RM3 for breeding (Special Diet Services, UK). Temperature (20–24 ^o^C) and relative humidity (45–65%) were maintained in a 12-hour light-dark environment.

All animal work was approved by the Welfare and Ethical Treatment of Animals Committee, under Home Office Project (PAFCCC160) and personal licences (I94242D3D, IDFA32717, I372C0F97) under the Animals (Scientific Procedures) Act 1986, as amended by EU Directive 2010/63/EU.

Every effort was made to ameliorate animal suffering. Animals were inspected regularly by trained and experienced staff, with 24-hour access to veterinary advice, and consideration was given to socialisation by allowing time for settling into new (experimental) groups. In addition, environmental enrichment was routinely added to cages in the form of red plastic tunnels or nests, chew sticks and diet was supplemented by sunflower seeds.

On study completion, animals were sacrificed by exposure to a rising concentration of CO _2_ and death confirmed by exsanguination, according to Schedule 1 of the Animals (Scientific Procedures) Act 1986.

### Experimental design

Adult female (>8 <22 weeks) mice were randomly allocated into control or experimental groups and allowed to adapt to their social setting for 7 d before study start. Cages were adjacent to each other on the same level of a ventilated rack, in the same room, for the study duration.

Neither animal staff nor experimenters were blinded to the identity of the mice or the experimental group in which they were placed, before, during or after the study.

Sample size: Although this work was considered preliminary in nature and the studies carried out as pilots, group sizes of n = 3–5 were used, following consideration of power calculations using G*Power, ^12^ with an effect size of 1.75 and power of 80%.

Data analysis: Dependencies of calculated tumour volumes versus time were analysed by non-linear regression using exponential growth and exponential decay functions ( GraphPad Prism v6.05 software, Graphpad, US). Alternative, open source, software – R – can be found at https://www.r-project.org. Rate constants in both functions were constrained to positive values to maintain consistency with the function name. Values for plateau parameters in exponential decay function were set to zero.

### Protocol

A375 human melanoma cells (ATCC: CRL-1619; RRID:CVCL_UD29) and 5555 murine melanoma cells ^13^^–^^15^ were grown as directed, with the latter subject to commercial murine pathogen testing (IDEXX Bioanalytics GmbH, Germany) and both to in-house mycoplasma testing (MycoAlert Mycoplasma Detection kit, Lonza Rockland, USA) before use. Passage number was recorded for each study.

Cell lines were harvested on the morning of the study, kept on ice and transferred to the animal facility for use within 1 h. All animal work was carried out in the sterile environment of a Tecniplast CS5 Evo Changing station.

Mice were weighed, fur removed on one/both flanks by electric shaver and placed individually in a red plastic inhalation chamber connected to an anaesthetic machine (Vet-Tech Solutions, Congleton, UK), to which was connected an anaesthetic maintenance tube running into the changing station. General anaesthesia was induced using an isoflurane (Piramal Critical Care, UK)/oxygen mixture in a Series 3 vapouriser (O _2_ flow rate 2l/min, isoflurane 3.5–4%) and maintained when the mouse was removed from the chamber by lying the animal on its front, snout placed just inside the end of the anaesthetic maintenance tube and the isoflurane/oxygen flow switched to the tube (O _2_ flow rate 1.5l/min, isoflurane 1.5–2.5%). Prepared cells (3.5–5 × 10 ^6^, 100 μl in DMEM (Thermofisher Scientific, UK) were taken up in a 1ml plastic syringe and injected subcutaneously (s.c.) to one or both flanks using a 25mm/23G needle [ *Optional: cells can be re-suspended in ECM (Sigma), diluted 1:1 with DMEM*]. This procedure routinely took <3 min. Immediately after injection, the mouse was returned to its home cage, placed on its front and monitored during recovery, which routinely took <5 min. [ *Optional: s.c. injection may be carried out immediately after removing mouse from the inhalation chamber, while still under general anaesthesia. However, particularly with immortalised human tumours, care must be taken to avoid self-injection; on safety grounds the maintenance anaesthesia route is strongly recommended.*]

In addition to routine welfare monitoring, mice were weighed and checked for tumour growth daily. Body weight was used in conjunction with a body scoring system. ^16^ Deviation from normal health, >10% body weight loss, or a body condition score of 2 or less was referred to the University Vet or Welfare Officer. If any animal appeared distressed, or a tumour ulcerated, the animal was removed from study and killed by a Schedule 1 method.

Once established, tumours were measured twice in two dimensions (maximum breadth and length) using digital calipers, by the same person to avoid interindividual variation. Treatment was also started at this point, mice receiving either vehicle or drug daily (p.o.).

Dabrafenib methanesulfonate (LC Laboratories, MA, USA) was prepared as a 6.3 mg/ml suspension in vehicle (0.5%(w/v) hydroxypropylmethylcellulose, 0.2%(v/v) Tween-80) after 10 min sonication in a water bath and administered daily (p.o.) at 5 ml/kg, and a dose of 31.5 mg/kg. This is equivalent to approximately 150 mg of dabrafenib base for a 70 kg human, ^17^ approximately half of the recommended daily dose ( https://dailymed.nlm.nih.gov/).

Trametinib (LC Laboratories, MA, USA) was prepared as a 0.07048 mg/ml suspension in vehicle (0.5%(w/v) hydroxypropylmethylcellulose, 0.5%(v/v) Tween-20) and administered daily (p.o.) at 5 ml/kg, and a dose of 0.3524 mg/kg. This is equivalent to 2 mg of trametinib for a 70 kg human, ^17^ which is the recommended daily dose ( https://dailymed.nlm.nih.gov/).

Tumour volume was estimated using the formula ((width*width)*length)/2. ^18^ Tumour length was also monitored, and mice in which tumour length reached 15mm (either individually or in total if tumours on both flanks) were sacrificed by a Schedule 1 method and blood, tissues and tumours harvested as appropriate for downstream analysis.

## Results

**Figure 1.**
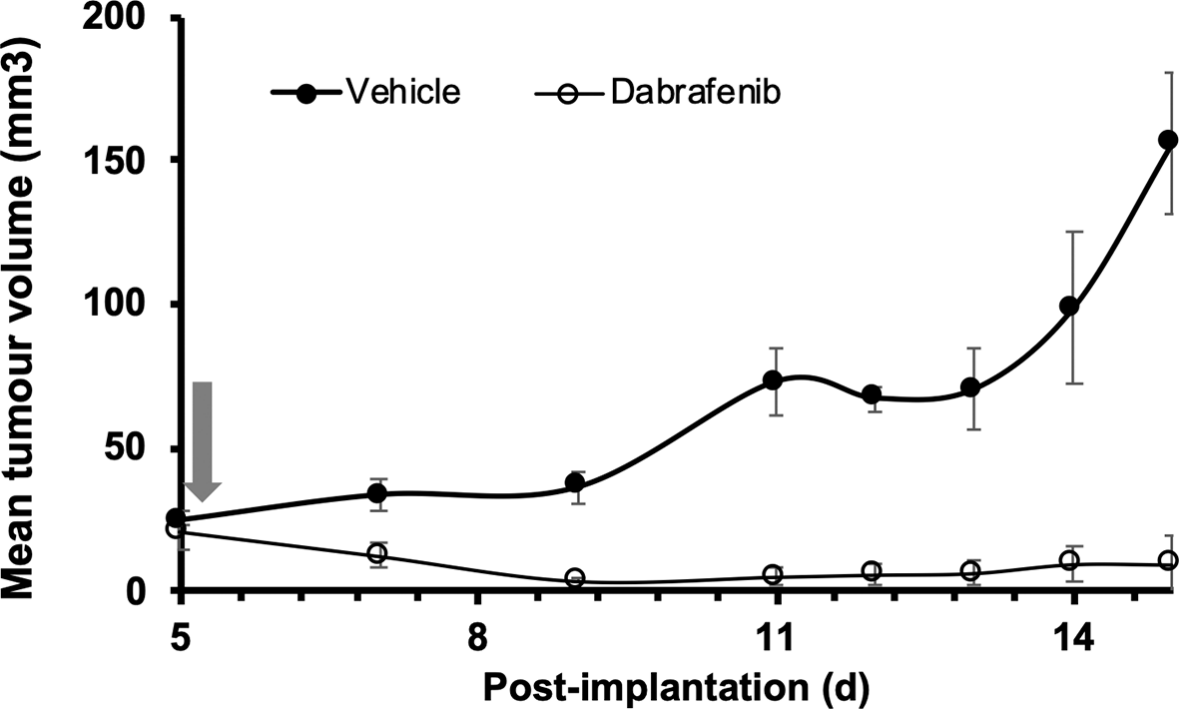
Growth of a murine melanoma syngeneic graft in 8HUM mice and response to dabrafenib treatment. Adult female 8HUM_Rag2 ^-/-^ mice (18–21 w, n = 5) were injected s.c. in one flank with 3.5 × 10 ^6^ 5555 murine melanoma cells, in 100 μl ECM diluted 1:1 with DMEM. Tumours were allowed to establish and on day five after implantation daily treatment was commenced with either vehicle (0.5% (w/v) hydroxypropylmethylcellulose, 0.2% (v/v) Tween 80; closed circles) or dabrafenib methanesulfonate (in vehicle, open circles) suspended at 6.3 mg/ml and administered at 5 ml/kg, such that dabrafenib dose administered was 31.5 mg/kg (arrow). Tumour measurements were taken three times weekly, then daily as required, and tumour volume calculated as detailed in Methods section. The study was terminated 15 days after implantation of cells. Data shown are mean tumour volume ± SEM.

8HUM mice were used to determine syngeneic growth of the murine melanoma cell line, 5555, derived from a C57BL/6_BRAF+/LSL-BRAFV600E;Tyr::CreERT2+/o transgenic model ^13^ and reported by Hirata *et al*. as being sensitive to the selective BRAF inhibitor, and vemurafenib precursor, PLX4720 ^19^ *in vitro,* but refractory to this drug *in vivo.* ^14^ More recently, the second-generation mutant BRAF inhibitor dabrafenib (in combination with the MEK inhibitor trametinib) has become standard of care in UK and Europe in the treatment of unresectable or metastatic BRAF ^V600^ mutant melanoma ( https://www.nice.org.uk/guidance/). ^20^ All data for this report are available online at. ^21^^,^^22^ As shown in Figure 1, 5555 cells were injected s.c. into one flank of adult female 8HUM mice, divided into two groups of five mice. After five days tumours had established in each animal and were measurable, at which point daily oral treatment with either vehicle or dabrafenib was started. While tumours in vehicle-treated mice continued to grow over the following two weeks, over the same period tumours in mice treated with dabrafenib became almost undetectable. These data demonstrate that C57BL/6-origin tumours can be grown in a syngeneic manner, and that the BRAF ^V600^ mutant murine melanoma cell line is exquisitely sensitive to the BRAF inhibitor dabrafenib.

**Figure 2.**
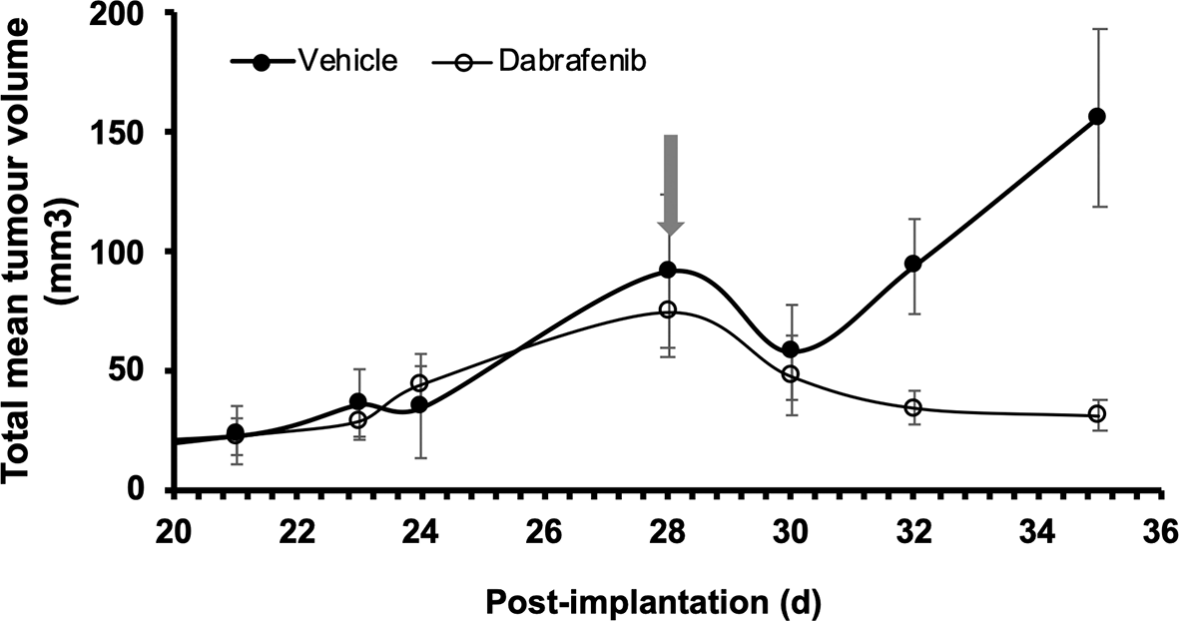
Response of A375 human melanoma xenograft to dabrafenib treatment in 8HUM_Rag2 ^-/-^ mice. Adult female 8HUM mice (11–19 w, n = 3) were injected s.c. in both flanks with 4.4 × 10 ^6^ A375 melanoma cells, in 100 μl DMEM. Tumours were allowed to establish and on day 28 after implantation daily treatment was commenced with either vehicle (0.5% (w/v) hydroxypropylmethylcellulose, 0.2% (v/v) Tween-80; closed circles) or dabrafenib (in vehicle, open circles) suspended at 6.3 mg/ml and administered at 5 ml/kg, such that dabrafenib dose administered was 31.5 mg/kg (arrow). Tumour measurements were taken three times weekly, then daily as required, and total volume of tumours on both flanks was calculated as detailed in Methods section. The study was terminated on d 35 after implantation of cells, although one vehicle-treated animal had to be removed from the study on d 29 as its total tumour size approached the maximum permitted under legislation. Data shown are mean tumour volume ± SEM.

By creating an immunodeficient variant of the 8HUM mouse line, where the *Rag2* locus is deleted, we extended work to xenografts with the BRAF mutant human melanoma cell line, A375. Figure 2 shows change in total mean tumour volume following s.c injection of A375 cells injected into both flanks of adult female 8HUM_Rag2 ^-/-^ mice. Daily treatment of these mice started 28 d after injection of cells, either with vehicle or dabrafenib (arrow); while the tumours in the former group continued to grow, tumours in the mice treated with the BRAF inhibitor shrank in size until by d35 (at which point the vehicle-treated mice had to be sacrificed due to tumour size) there was a significant difference in the treatment effect between the two groups ( Figure 2). The data from vehicle group follows the exponential growth dependency and does not fit with the exponential decay function. Conversely, the dabrafenib group data follows exponential decay dependency and does not fit the exponential growth function. These data clearly demonstrate not only that it is possible to grow a human melanoma cell line as a xenograft in this immunodeficient version of the 8HUM mouse model, but it is also possible to demonstrate sensitivity of A375 tumours to BRAF inhibitors.

**Figure 3.**
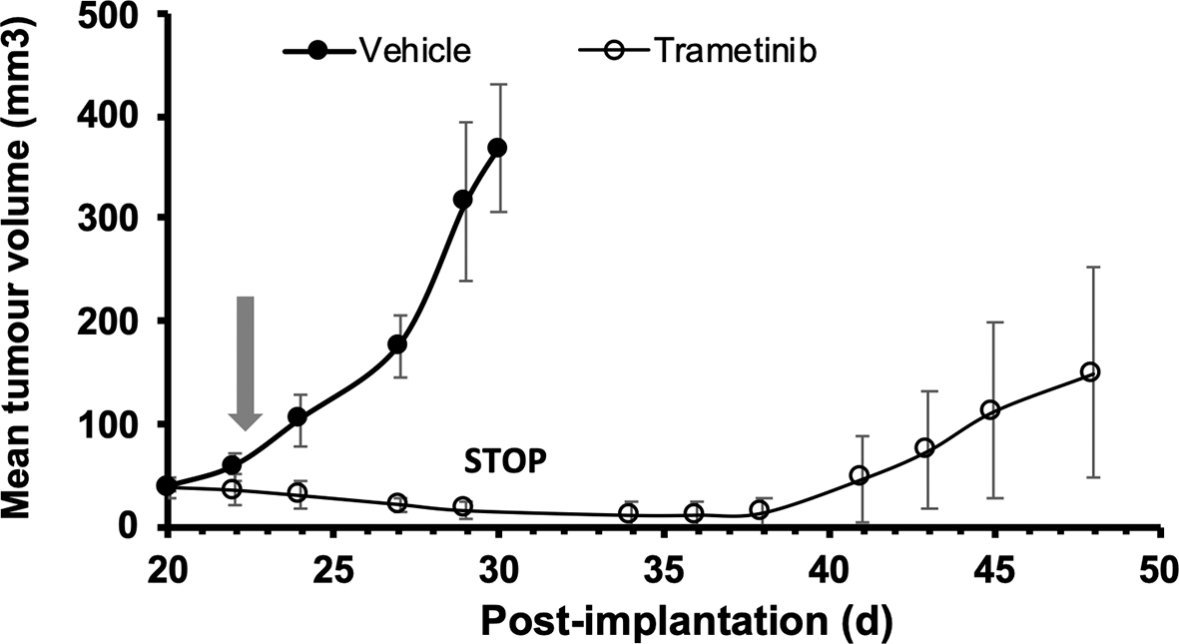
Response of A375 human melanoma xenograft to trametinib treatment in 8HUM_Rag2 ^-/-^ mice. Adult female 8HUM mice (8–18w, n = 3 or 4) were injected s.c. in one flank with 5 × 10 ^6^ A375 melanoma cells, in 100 μl DMEM. Tumours were allowed to establish and on day 22 after implantation daily treatment was commenced with either vehicle (0.5% (w/v) hydroxypropylmethylcellulose, 0.2% (v/v) Tween 80; closed circles) or trametinib (in vehicle, open circles) suspended at 0.07 mg/ml and administered at 5 ml/kg, such that trametinib dose administered was 0.35 mg/kg (arrow). Tumour measurements were taken three times weekly, then daily as required, and tumour volume calculated as detailed in Methods section. Vehicle-treated mice were sacrificed on d 30 after implantation of cells; trametinib treatment was discontinued (STOP) at that time for the drug-treated group to determine whether tumour re-growth would occur in the absence of drug. Data shown are mean tumour volume ± SEM.

Dabrafenib is used in a clinical setting in combination with the MEK inhibitor trametinib. We tested the ability of trametinib to stop tumour growth, using A375 cells injected s.c. in the flank of adult female 8HUM_Rag2 ^-/-^ mice ( Figure 3). Daily treatment with vehicle or drug commenced on d 22 after cell injection (arrow), and in the following period tumours in mice treated with vehicle continued to grow until by d 30 they had reached the maximum size permitted. At this point ( Figure 3, STOP) tumours in the 8HUM_Rag2 ^-/-^ mice had regressed to the point where they were essentially undetectable, and trametinib treatment was stopped. Interestingly, over the following 10 days, tumours began to regrow in the absence of treatment until they were again palpable and measurable, and continued to grow over the next week or so until the study was terminated on d 48 ( Figure 3).

## Discussion and conclusions

In a previous publication ^6^ we have demonstrated the utility of the 8HUM model in better predicting human drug metabolism and disposition, and in the current report show how the 8HUM mouse and its immunodeficient variant 8HUM_Rag2 ^-/-^ are capable of hosting both syngeneic tumours and xenografts, respectively.

While numbers of animals used in syngeneic or xenograft work in pre-clinical drug development are difficult to assess, the total will be significant given the number of drug candidates being tested across Pharma at any given time, and such growth of tumours *in vivo* is also carried out in other research areas, for example, toxicology. A PubMed search for papers published in 2019 found ~5,000 papers containing ‘xenograft’ in the title or abstract, illustrating the extent to which the 8HUM and 8HUM_Rag2 ^-/-^ models – modified as appropriate by gene editing to recapitulate disease models - may be able to address 3Rs issues by both **refinement**: generation of better, more human-relevant data, and **reduction** – use of fewer animals without loss of statistical power. Pre-clinical use of humanised models to prevent failure of a drug candidate during clinical testing because of species differences in drug disposition would undoubtedly save significant numbers of mice. They will also allow complex drug combinations to be tested and treatment regimens optimised in a manner which is not feasible by clinical trial and reduce the chances of drug-drug interactions.

The 8HUM has some limitations, and it should be noted that a minor complement of murine P450 enzymes remain, and that the Phase II enzymes are murine. These may potentially contribute to drug disposition, as may other pathways e.g., drug transporters. However, the advent of CRISPR/Cas9, as used here to delete the Rag2 locus in the 8HUM mouse, means that it should be relatively simple to additionally modify the 8HUM model to further enhance versatility.

## Author contributions

Conceptualization    Wolf, Henderson, Kapelyukh

Data Curation      McLaren, Henderson, Kapelyukh

Formal analysis     Kapelyukh, Henderson

Funding acquisition    Wolf, Henderson

Investigation      Kapelyukh, McLaren, Henderson

Methodology      Wolf, Kapelyukh, McLaren, Henderson

Project Administration  McLaren, Henderson

Resources       McLaren, Henderson

Supervision      Wolf, Henderson

## Data availability

### Underlying data

Figshare: Underlying data for ‘Improving the predictive power of xenograft and syngeneic anti-tumour studies using mice humanised for pathways of drug metabolism’. https://doi.org/10.6084/m9.figshare.20060465.v1. ^21^

This project contains the following underlying data:

• Data file 1: FIG 1 BRI995 Weights&Tumours 280219.xlsx
• Data file 2: FIG 2 BRI1102 Weights&Tumour 010421.xlsx
• Data file 3: FIG 3 BRI1119 Weights&Tumour 210621.xlsx

### Reporting guidelines

Figshare: ARRIVE checklist for ‘Improving the predictive power of xenograft and syngeneic anti-tumour studies using mice humanised for pathways of drug metabolism’. https://doi.org/10.6084/m9.figshare.20060465.v1. ^22^

Data are available under the terms of the Creative Commons Attribution 4.0 International license (CC-BY 4.0)
