# Supplementary material for: Improving the predictive power of xenograft and syngeneic anti-tumour studies using mice humanised for pathways of drug metabolism
Source: F1000Res. 2023 Mar 27;11:1081. Originally published 2022 Sep 21. [Version 2] doi: 10.12688/f1000research.122987.2 (PMC10090862; doi:10.12688/f1000research.122987.2)
Supplement: Peer review history

# Peer review: Reviewer response for version 2

**DOI:** 10.5256/f1000research.145248.r167839

**Published:** 2023-04-11

**Authors:** Chris Goldring

**Affiliations:** 1 MRC Centre for Drug Safety Science, Department of Molecular and Clinical Pharmacology, University of Liverpool, Liverpool, UK

I would consider that the authors responses are satisfactory, and would be satisfied that this work is now acceptable for indexing.

Is the work clearly and accurately presented and does it cite the current literature?

Yes

If applicable, is the statistical analysis and its interpretation appropriate?

Yes

Are all the source data underlying the results available to ensure full reproducibility?

Yes

Is the study design appropriate and is the work technically sound?

Yes

Are the conclusions drawn adequately supported by the results?

Partly

Are sufficient details of methods and analysis provided to allow replication by others?

Yes

Reviewer Expertise:

NA

I confirm that I have read this submission and believe that I have an appropriate level of expertise to confirm that it is of an acceptable scientific standard.

# Peer review: Reviewer response for version 2

**DOI:** 10.5256/f1000research.145248.r167840

**Published:** 2023-03-29

**Authors:** Russ Prough

**Affiliations:** 1 Hepatobiology&Toxicology Program, University of Louisville, Louisville, KY, USA

The Authors have revised the areas of my concern and I find it acceptable for indexing.

Is the work clearly and accurately presented and does it cite the current literature?

Yes

If applicable, is the statistical analysis and its interpretation appropriate?

Yes

Are all the source data underlying the results available to ensure full reproducibility?

Yes

Is the study design appropriate and is the work technically sound?

Yes

Are the conclusions drawn adequately supported by the results?

Yes

Are sufficient details of methods and analysis provided to allow replication by others?

Yes

Reviewer Expertise:

drug metabolism, CYPs

I confirm that I have read this submission and believe that I have an appropriate level of expertise to confirm that it is of an acceptable scientific standard.

# Peer review: Reviewer response for version 1

**DOI:** 10.5256/f1000research.135046.r160109

**Published:** 2023-02-13

**Authors:** Chris Goldring

**Affiliations:** 1 MRC Centre for Drug Safety Science, Department of Molecular and Clinical Pharmacology, University of Liverpool, Liverpool, UK

This is an important preliminary report that will be of value to the academic and pharmaceutical drug development scientists. 

 Some specific points:

 Methodologically, this looks to be a preliminary but robust report. The lack of characterisation of the tumours post-mortem is an issue that the authors could address using conventional histological characterisation. This is not a request that should not significantly impede time to publication, but would add more depth to the findings.

 In the abstract –“The poor predictive power of animal models in pre-clinical studies is predominantly due to lack of efficacy or safety reasons, which in turn can be attributed mainly to the significant species differences in drug metabolism between humans and animals.” This is largely correct for older traditional investigative compounds, however as drug development pipelines have evolved, especially with the advent of more large molecule development, the issue of target-drug conservation of pharmacodynamic effect becomes very relevant. The authors should make some account of this (given that the two agents looked at in this report are conventional small molecule inhibitors).

 Pharma largely uses rat models, for various reasons. The authors should note this in the context of their own mouse model.

Is the work clearly and accurately presented and does it cite the current literature?

Yes

If applicable, is the statistical analysis and its interpretation appropriate?

Yes

Are all the source data underlying the results available to ensure full reproducibility?

Yes

Is the study design appropriate and is the work technically sound?

Yes

Are the conclusions drawn adequately supported by the results?

Partly

Are sufficient details of methods and analysis provided to allow replication by others?

Yes

Reviewer Expertise:

NA

I confirm that I have read this submission and believe that I have an appropriate level of expertise to confirm that it is of an acceptable scientific standard, however I have significant reservations, as outlined above.

# Response

**Published:** 2023-03-06

**Authors:** Colin Henderson

**Affiliations:** Univesrity of Dundee, UK

*Point 1: Pathological characterisation*

 It should be noted that the xenografts used in this study were derived from a human cell line and not from a solid tumour. In addition, tumour samples are not available for the drug treated groups because of the effectiveness of the treatments used. On this basis we feel adding histopathological data would add little to the main conclusions of the study. 

*Point 2: Species differences in drug metabolism remain a significant impediment in drug development*

 In the light of the Reviewer’s comment, we have changed the sentence to read "Species differences in drug metabolism can be a major impediment to drug discovery programmes, for example in the demonstration of drug efficacy as a consequence of high rates of metabolism in rodents. Furthermore, the spectrum of metabolites produced, which can be pharmacologically active, is often significantly different between humans and rodents. These factors can confound the extrapolation of animal data to man particularly when drug combination therapies are being tested”. 

*Point 3:*

 The Reviewer is correct to point out that both rats and mice are used by industry in the drug development process. However, the utility of mice for growing xenografts, including patient-derived xenografts, and the development of ‘mouse clinical trials’, along with the more advanced use of mice in genetically engineered models (such as 8HUM) means that increasingly mice are being considered more useful for drug development, especially in oncology. A sentence to this effect has been placed in the Discussion, and additional references added.

# Peer review: Reviewer response for version 1

**DOI:** 10.5256/f1000research.135046.r159796

**Published:** 2023-01-19

**Authors:** Russ Prough

**Affiliations:** 1 Hepatobiology&Toxicology Program, University of Louisville, Louisville, KY, USA

The manuscript in general is clearly written and leads the reader through the results. The statistical details providing valid conclusions is well documented. The Authors have developed a unique murine model to provided data on human metabolism. Past reports document the utility of model as being useful to document. A couple specific questions arise:

 1.  If the authors had used the original mouse line (with the Rag2 gene present), would the results have been the same or less sensitive to the anti-cancer drugs? 

 2.  If that data exists, has it been published? If not would inclusion of such data strengthen the current manuscript. This would document the importance of the Rag2 KO.

 3.  Some anti-cancer drugs induced their own metabolism. Has this been studied for Dabrafenib or Tremetinib?

Is the work clearly and accurately presented and does it cite the current literature?

Yes

If applicable, is the statistical analysis and its interpretation appropriate?

Yes

Are all the source data underlying the results available to ensure full reproducibility?

Yes

Is the study design appropriate and is the work technically sound?

Yes

Are the conclusions drawn adequately supported by the results?

Yes

Are sufficient details of methods and analysis provided to allow replication by others?

Yes

Reviewer Expertise:

drug metabolism, CYPs

I confirm that I have read this submission and believe that I have an appropriate level of expertise to confirm that it is of an acceptable scientific standard, however I have significant reservations, as outlined above.

# Response

**Published:** 2023-03-06

**Authors:** Colin Henderson

**Affiliations:** Univesrity of Dundee, UK

*Point 1: If the authors had used the original mouse line (with the Rag2 gene present), would the results have been the same or less sensitive to the anti-cancer drugs?*

 The human cell line (A375) would not have grown in an immunocompetent 8HUM line.

*Point 2:  If that data exists, has it been published? If not would inclusion of such data strengthen the current manuscript. This would document the importance of the Rag2 KO.*

 Data does not exist for the reason outlined above.

*Point 3: Some anti-cancer drugs induced their own metabolism. Has this been studied for Dabrafenib or Trametinib?*

 Dabrafenib is principally metabolised by CYP2C8 and CYP3A4, and in common with many drugs, can induce its own metabolism (see - Clinical Pharmacokinetics and Pharmacodynamics of Dabrafenib, (2019) Puszkiel, A. et al., PMID: 30094711). With regard to Trametinib, the position is less clear. We have also been able to reproduce the known induction of P450 CYP3A4 by dabrafenib in the humanized mice, further demonstrating the utility of the model in anticancer drug development (unpublished data), and a sentence to this effect has been added to the text of the Results section.
